# Multi-Level Social Health Insurance System in the Age of Frequent Employment Change: The Urban Unemployment-Induced Insurance Transition and Healthcare Utilization in China

**DOI:** 10.3390/healthcare7020077

**Published:** 2019-06-13

**Authors:** Bocong Yuan, Jiannan Li, Lily Wu, Zhaoguo Wang

**Affiliations:** 1 Center for Tourism Development Planning and Research, School of Tourism Management, Sun Yat-sen University, West Xingang Rd. 135, Guangzhou 510275, China; yuanbc@mail.sysu.edu.cn (B.Y.); wull9@mail2.sysu.edu.cn (L.W.); 2 International School of Business & Finance, Sun Yat-sen University, West Xingang Rd. 135, Guangzhou 510275, China; 3 School of Economics and Management, Shenyang Agricultural University, Shenyang 110041, China

**Keywords:** health insurance transition, unemployment, urban employee basic medical insurance (UEBMI), urban resident basic medical insurance (URBMI), new cooperative medical scheme (NCMS), healthcare utilization, Gig Economy

## Abstract

Job tenure has been significantly shortened with the prevalence of the gig economy around the world. Workers are faced with a new age of frequent employment change. This emerging situation is out of expectation of social health insurance policymakers. As the multi-level social health insurance system in China is closely associated with employment status; urban workers cannot enjoy the urban employee basic medical insurance (UEBMI) during the unemployment period. At this time, unemployed rural-to-urban migrant workers can only rely on the new cooperative medical scheme (NCMS) and unemployed urban residents can only rely on the urban resident basic medical insurance (URBMI). This study provides a preliminary analysis on healthcare utilization change triggered by the unemployment-induced social health insurance transition that has never been investigated. Using the data of a nationwide survey, empirical results show that the unemployment-induced social health insurance transition can significantly deteriorate the healthcare utilization of insurance beneficiaries experiencing the transitions from the UEBMI to the NCMS (or from the UEBMI to the URBMI). Specifically, the outpatient service quality and the conventional physical examination become worse, and the out-of-pocket expenditure increases. Therefore, the multi-level social health insurance system currently in effect can expose workers to a high risk of insufficient health security in the age of frequent employment change.

## 1. Introduction

A growing number of enterprises and institutions in China are adapting to the trend of gig economy and tending to hire an increasing number of temporary staff instead of permanent staff. Reports published by LinkedIn, a famous business-oriented social networking service, show that the average job tenure has reduced to 22 months in China [1], and the average tenure for the first job has even reduced to seven months for the post-1995s generation [2]. Workers are faced with a new age of frequent employment change, which they have never experienced before. For policy makers of social health insurance, this emerging situation is out of expectation. It may produce a serious challenge to the existing multi-level social health insurance system in China that is closely associated with employment status.

In contrast with the United States, China’s health insurance system is based on social health insurance provided by the state [3]. The commercial health insurance market in China is still far from maturity and cannot fully supplement social health insurance for residents [3]. Therefore, China’s social health insurance largely determines the level of healthcare services that residents can enjoy. China’s public healthcare services are covered by the multi-level social health insurance system, which includes the urban employee basic medical insurance (UEBMI) for urban employed population or retirees, the urban resident basic medical insurance (URBMI) for urban unemployed population, and the new cooperative medical scheme (NCMS) for the rural population [4].

Urban workers cannot enjoy the urban employee basic medical insurance (UEBMI) as before once they lose their jobs [5,6]. During the unemployment period, rural-to-urban migrant workers have to rely on the new cooperative medical scheme (NCMS), while regular urban residents have to rely on the urban resident basic medical insurance (URBMI) [5,6]. There is a significant difference between these social health insurance systems in the payment rate, the coverage of insurance, the level of treatment, and the reimbursement rate. The UEBMI is obviously superior to the URBMI and the NCMS in these aspects. For example, in terms of the average reimbursement rate, it is around 70% for the UEBMI beneficiaries, but only about 50% for the URBMI and the NCMS beneficiaries [5,6]. In this case, the UEBMI will to a greater extent help alleviate the burden of out-of-pocket expenditure and improve access to high-quality health services. Therefore, the unemployment-induced insurance transition from the UEBMI to the URBMI or NCMS would damage the healthcare utilization, with reducing access to high-quality healthcare services and increasing the cost of healthcare services.

Further, for workers experiencing the unemployment-induced insurance transition from the UEBMI to the NCMS during the employment change period, the damage to their healthcare utilization appears more serious. Due to the great rural-urban disparity in healthcare access [7,8,9], the NCMS beneficiaries generally have worse access to healthcare services than the URBMI beneficiaries, despite also being the victim of losing the coverage of UEBMI. Moreover, the dependency principle of a social health insurance system can deter NCMS beneficiaries from seeking healthcare service in urban areas [10,11,12]. The NCMS beneficiaries cannot have their health expenditure covered unless they return to hometown to seek healthcare service in designated hospitals [13,14,15,16]. In this case, the transport and other costs caused by the round trip to hometown are usually beyond the NCMS benefits [17]. Hence, they are faced with the dilemma of seeking healthcare services in urban area, where they have to bear high out-of-pocket expenditure, and returning to their hometown for healthcare services where the NCMS benefits cannot cover the round-trip costs [18,19]. As a result, for rural-to-urban migrant workers, the unemployment-induced insurance transition from the UEBMI to the NCMS can lead to the greater damage to their healthcare utilization.

This study provides a preliminary investigation on the healthcare utilization change triggered by the unemployment-induced insurance transition that has never been explored. This issue arises from the prevailing gig economy around the world. Now, it is becoming increasingly difficult to acquire long job tenure. A growing number of people have to bear the frequent employment change and the resulting frequent gap between jobs. The temporary unemployment status is thus becoming an increasingly common experience in one’s life. However, the current health insurance systems are not ready for the change of employment environment. The link-up of health insurance systems is not well established enough to respond to the unemployment-induced health insurance transition. As such, the frequent unemployment status can inevitably cause the damage to the healthcare utilization. To better understand this issue, it is essential to empirically examine the effect of the unemployment-induced health insurance transition on healthcare utilization in the context of frequent employment change.

Moreover, this issue is of great significance in China. Compared with developed countries, the commercial health insurance market in China is not yet mature. It has difficulty, at the present time, becoming a reliable and widely-accepted solution for unemployed people for maintaining a level of healthcare utilization. More importantly, due to being closely associated with employment status, Chinese multi-level social health insurance system can trigger passive health insurance transition in the context of frequent employment change, making people in temporary unemployment status not entitled to benefits of health insurance with wider coverage, but have to turn to others with narrower coverage. Thus, it is particularly necessary to investigate how this issue presents in China.

This paper is structured as follows. In the following sections, this paper first describes the foundation laid by previous research, and the motivation for initiating this study. Then, this paper discusses the status quo of China’s social health insurance system, and based on this, analyzes the potential effect of unemployment-induced insurance transition on health utilization in the context of frequent employment change. In the following methods section, this paper uses the data of a nationwide survey (China health and retirement longitudinal study, CHARLS) to empirically examine the effect of health insurance transition from the UEBMI to URBMI or NCMS on health utilization. Meanwhile, this paper makes a detailed description of measures of variables and offers the technical details to clarify the reasonability of the heterogeneously robust linear regression approach applied in this study. Finally, in the discussion and conclusion sections, this study concludes that the current social health insurance system in China has not coped well with the emerging problem of frequent insurance transition triggered by the frequent employment change. The rural-to-urban migrant workers who experience the transition from UEBMI to NCMS are found suffering a far more severe deterioration of healthcare utilization than regular urban residents who experience the transition from UEBMI to URBMI. The current social health insurance schemes thus need to be integrated to meet the new requirements. Future research directions and implications of the present research have also been discussed.

## 2. Literature Review

### 2.1. The Motivation of the Theme

Previous research has laid a solid foundation for this study. Some of them focus on one kind of social health insurance to explore its effect on health outcomes (e.g., [20,21]) or to make a comparison between before and after its implementation (e.g., [22]), some focus on the specific provinces or cities, or specific groups to examine the influence they get from the current social health insurance (e.g., [23,24]). Also, a few of research provides an overview of the current social health insurance (e.g., [25]), and compares the difference between the different kinds of it (e.g., [26]), and some others concern the problems generated by this system, such as moral hazards, adverse selection (e.g., [27]), or benefit distribution (e.g., [28]). These devoted efforts help better understand the actual role of the current social health insurance and the design of this system itself. However, with the phenomenon of frequent employment change emerging in Chinese labor market, the findings of previous studies cannot be used to explain the new issue of passive unemployment-induced social health insurance transition and its health outcomes. Thus, it is required to give an empirical investigation on this never explored issue and, in doing so, helps improve the development of China’s multi-level social health insurance system.

### 2.2. The Status Quo of Social Health Insurance in China

To briefly summarize the differences between UEBMI, URBMI and NCMS it can help to better understand the problem of unemployment-induced social health insurance transition.

(1) First, the target population is different.

The UEBMI mainly benefits the urban employed [29], while the URBMI mainly serves unemployed people who have urban household registration, and the NCMS is designed for people with rural household registration [30,31].

(2) Second, the payment requirements and funding source are different.

The UEBMI is mandatory for the urban employed and jointly paid by employers and employees [32], while the URBMI and NCMS are both voluntary [33,34,35,36] and enjoy government subsidies on the basis of individual contribution (plus collective contribution for the NCMS) [30]. Proportional to personal wages, the level of payment for UEBMI is generally higher than that for the UEBMI and NCMS [32]. The UEBMI sets a minimum length of time for payment. For those having reached it, there is no need for further payment after retirement [37]. Instead, the URBMI and NCMS need to be paid annually without any predetermined length of time.

(3) Third, the coverage level of treatment is different.

Since the level of funding for URBMI and NCMS are relatively lower, the general level of their medical treatment is shown lower than that of UEBMI’s [29,38]. The drug reimbursement list of UEBMI is extensive [39,40], whereas that of NCMS and URBMI is relatively fragmented and varies across different provinces, since many provinces create their own separate and smaller list [41]. For the higher level of benefits, UEBMI beneficiaries are found more likely to take medicines than URBMI and NCMS beneficiaries [42].

(4) Finally, the reimbursement level is different.

The reimbursement rate and reimbursement cap enjoyed by URBMI and NCMS beneficiaries are lower than that for UEBMI [29,32,43,44,45]. Moreover, the reimbursement rate for NCMS is higher in rural township hospitals than in municipal or above hospitals, and the reimbursement procedure in municipal or above hospitals is more complicated for NCMS beneficiaries [46]. All of this causes a barrier for NCMS beneficiaries seeking better healthcare services [10,47,48]. More details are provided in Table 1.

### 2.3. Unemployment-Induced Insurance Transition and Health Utilization in the Context of Frequent Employment Change

As mentioned above, the UEBMI is superior in many aspects to the URBMI and the NCMS. For the UEBMI, its stable and reliable funding source can ensure its higher funding level, which in turn can support its high level of treatment coverage for the beneficiaries [43,50]. In term of expenditure on healthcare services provided by municipal or above hospitals, the UEBMI sets a higher reimbursement rate and a higher reimbursement cap line than the other two insurance systems do, which not only alleviates the financial burden of medical expense by the beneficiaries, but also encourages access to the high quality of healthcare services [43,51]. Therefore, the unemployment-induced insurance transition from the UEBMI to the URBMI or NCMS can damage the healthcare utilization (e.g., the decrease in access to the high-quality healthcare services, and the increase in the out-of-pocket expenditure of healthcare services). Further, compared with the transition from the UEBMI to the URBMI, the transition from the UEBMI to the NCMS can further deteriorate the healthcare utilization of people, since the coverage scope is much narrower for NCMS beneficiaries [52,53,54]. Besides, the lower reimbursement rate in municipal hospitals compared to township hospitals can deter the NCMS beneficiaries from seeking better healthcare services. The unmet inpatient need has gone up to 27.9% for NCMS beneficiaries until 2008 [49], and still been great until now.

## 3. Materials and Method

### 3.1. Study Design

The data of China health and retirement longitudinal study (CHARLS-2015) is used in this study. CHARLS is a widely-used nationwide survey using stratified random sampling, and it collects a high-quality and nationally representative sample of Chinese residents over the age of 45. This survey is jointly initiated by the Center for Social Science Survey in China and the Youth League Committee of Peking University, and is implemented by the National Development Institute of Peking University. CHARLS-2015 is made public since 2017. This survey covers 150 counties, including 450 communities and villages across 28 provinces and municipalities in China, among which about 52.6% are rural areas and 47.4% are urban areas. The sample has covered about 12,400 households in total. This population surveyed is representative of people who are more vulnerable to diseases and more sensitive to the social health insurance transition.

### 3.2. Variables

In this study, three critical dependent variables reflecting healthcare utilization (i.e., outpatient service quality, conventional physical examination and outpatient service cost) are examined. Respondents in this study are those who are the insured of UEBMI in 2013, but turn out to be uninsured during 2013–2015 because of unemployment (indicated as UEBMI: Yes_2013_→No_2013–2015_). According to the insurance status of NCMS in 2013 and during 2013–2015 for unemployed rural-to-urban migrant workers, four conditions are tested as independent variables (that is, NCMS: Yes_2013_→Yes_2013–2015_, Yes_2013_→No_2013–2015_, No_2013_→Yes_2013–2015_, and No_2013_→No_2013–2015_). The similar four combinations of insurance status of URBMI in 2013 and during 2013–2015 for unemployed urban residents are also tested as independent variables (URBMI: Yes_2013_→Yes_2013–2015_, Yes_2013_→No_2013–2015_, No_2013_→Yes_2013–2015_, and No_2013_→No_2013–2015_). More details about dependent and independent variables have been shown in Table 2.

Gender, age and the chronic disease history that may affect healthcare utilization have been controlled in the regression analysis. The chronic disease history includes hypertension, dyslipidemia, diabetes, cancer, chronic lung diseases, liver disease, heart disease, stroke, kidney disease, stomach or other digest disease, emotional or psychiatric problem, memory related disease, arthritis or rheumatism, and asthma. More details about demographic variables and the chronic disease history have been shown in Table 3.

### 3.3. Analytic Strategy

The dataset used in this study is merged from three different data subsets: “healthcare and insurance”, “health status and functioning” and “demographic background”, according to “individual identity (ID)”. The unmatched observations are dropped. The statistical software Stata 13.1 is used in the data analysis. The heterogeneously robust linear regression is applied in this study. Standard errors have been clustered on community level and household level, respectively. The advantage of this method lies in that the estimates can still remain consistent and efficient when individuals could not comply with the independent and identically distributed (I.I.D.) condition. Considering respondents from the same community (or household) are more likely to be homogeneous, the heterogeneously robust linear regression would compute the robust standard errors clustered on community (or household) level instead of the common one to judge the statistical significance. In this way, the estimates are reliable.

## 4. Empirical Results

Results of Table 4 show that the unemployment-induced social health insurance transition would have negative effect on healthcare utilization. The unemployment-induced transition of UEBMI from active to inactive status would lead to the deterioration of healthcare utilization, which is manifested as the decline of outpatient service quality, the decrease in the number of items of the purchased conventional physical examination, and the increase in out-of-pocket expenditure. What is worth noticing is that such deterioration still exists and is significant, even though people are insured with the NCMS (−1.1027 and −0.8709, outpatient service quality; −3.3827 and −2.1574, conventional physical examination; 0.1596 and 0.1448, out-of-pocket expenditure, all above with *p* < 0.05 for people in the condition of UEBMI: Yes→No and NCMS: Yes→Yes and UEBMI: Yes→No and NCMS: No→Yes respectively).

Similar empirical results have been found for the transition between UEBMI and URBMI (see Table 5), although the deterioration of healthcare utilization is slighter than that between UEBMI and NCMS. Specifically, the outpatient service quality and physical examination do not significantly become worse for unemployed urban residents whose URBMI is from inactive to active (UEBMI: Yes→No and URBMI: No→Yes). Besides, the outpatient service quality and out-of-pocket expenditure do not get significantly worse for unemployed urban residents whose URBMI is from inactive to inactive (UEBMI: Yes→No and URBMI: No→No).

## 5. Discussion

It is generally known that job tenure has been significantly shortened with the prevalence of the gig economy in China, and a long-termed job has become less available. This new situation leaves the social health insurance system in force lagging behind the status quo of China’s economy. The mismatch between the continuing healthcare demand and the health insurance transition during the short unemployed gap period starts to emerge. The current social health insurance system does not cope well with this problem.

In the past, the multi-level social health insurance system is considered to have well covered different types of population. The employed people are covered by UEBMI, and the unemployed people can also enjoy health insurance of URBMI and NCMS. The employment change is not so frequent as before, and the social health insurance system in force does not take the employment change as a matter that frequently happens. Neither the URBMI nor the NCMS is particularly designed for people that experience short unemployed gap periods. Thus, the frequent social health insurance transition resulting from the employment change brings not only the great managing difficulty for beneficiaries, but also huge administrative costs for administrative agencies.

Moreover, the conflict between the continuing demand for healthcare utilization and the passive social health insurance transition can lead to severe consequences. For example, continuing healthcare is particularly in need for chronic disease patients. However, during the short unemployed gap period, the healthcare utilization can be suppressed, and some necessary treatments have to be broken off for saving out-of-pocket expenditure. As such, the patients have to adjust their treatment demand to make it covered by the transitional social health insurance rather than in accordance with their illness status. Even though people can restore their beneficiary status of UEBMI after getting the next job, the interruption or suppression of healthcare utilization within the short unemployed gap period can still have serious impact on chronic disease patients.

Further, the rural-to-urban migrant workers are more vulnerable to the social health insurance transition. Although the healthcare utilization is also damaged for regular urban residents that experience the transition from UEBMI to URBMI, the deterioration of healthcare utilization is much more severe for rural-to-urban migrant workers who experience the transition from UEBMI to NCMS. The difference in benefit package between the two schemes (URBMI and NCMS) can partially lead to this situation. And also, the over-concentration of healthcare resources and the resulting great rural-urban disparity in healthcare access can be the culprit of this asymmetry.

## 6. Conclusions

This study is the first attempt to assess the impact of health insurance transition induced by employment change on healthcare utilization. It adds important new knowledge to the existing empirical evaluation of health insurance system. Results further provide empirical support to the idea that the emerging gig economy and the resulting frequent employment change can produce a new challenge to the social health insurance system in China. For policy makers of social health insurance, it is necessary to establish a proper transferring mechanism to integrate UEBMI, URBMI and NCMS. This mechanism needs to link with the original health insurance system, whereby the barrier to receiving NCMS reimbursement for most of rural-to-urban migrant workers could be well dealt with. To successfully build this mechanism, the cohesion between different social health insurance schemes needs to be strengthened through solving some problems like payment age conversion, medical funding compensation for local areas. Moreover, it is helpful to construct an integrated health insurance information platform that implements the centralized management of health information and compensation schemes of various diseases for all kinds of the insured. For those without sufficient conditions for integration of different social health insurance schemes, this practice can serve as the first move towards this integration. Further, the urban-rural dual structure of current social health insurance in China is designed for ease of administration. However, the separate healthcare administration for different social health insurance schemes has now hindered the resolution of emerging issues. Thus, it is essential to unify the administration to facilitate the integration of health insurance, and also to alleviate the financial pressure of operation. Finally, for now, the mitigation measure is to set the buffer period, for example, by 12 months since unemployment, during which the entitlement to UEBMI benefits can be retained. This effort may minimize the damage to health utilization during the period of health insurance transition.

This study is not free of limitations. The nationwide survey used in this study simply documents the information of health insurance transition during the year of 2013–2015, which cannot be used to reveal the dynamics of its effect on health outcomes in a long term. With the subsequent data gradually made public, the effect of a health insurance transition on health outcomes can get the dynamic monitoring. Thus, as more relevant data become available, future research can use new data to provide a comprehensive exploration of this issue.

## Figures and Tables

**Table 1:** A summary of differences between UEBMI, URBMI and NCMS.

Index	Multi-Level Social Health Insurance System in China			Remarks
	UEBMI	URBMI	NCMS	
Target population	Urban employed population, including employed by organizations, self-employed and retirees [29].	Unemployed people with urban household registration, including unemployed elderly residents, low-income people living on basic living allowances, severely disabled people, students and children, etc. [30,31].	People with rural household registration, including rural-to-urban migrant workers, and regular rural residents [30,31].	The most generous UEBMI covers 15% and the most straitened NCMS covers over 68% populations [49].
Funding source	Jointly paid by employers and employees, without government subsidies [30].	With support of modest government subsidies on the basis of personal contribution [30].	Funded by individuals, collectives and the government [30].	
Payment requirements	Mandatory [33,34,35,36].	Voluntary [33,34,35,36].	Voluntary [33,34,35,36].	
	With a minimum length of time for payment (25 years for males and 20 years for females), and there is no need for further payment after retirement if the length of time is reached [37].	Without a minimum payment period and paid annually.	Without a minimum payment period and paid annually.	
	The level of payment is related to personal wages with the employer and employee undertaking 8% and 2% of the wage level, respectively [32].	The level of payment mainly relies on individual contributions, with government subsidies up to 36% [47].	The payment is based on the economic level of provinces and cities; some kinds of common payment level are CNY 100, CNY 200, CNY 300, CNY 400, or CNY 500 per year.	The level of payment for UEBMI is generally higher than that for URBMI and NCMS [32].
Coverage level of treatment	Its drug coverage is always more extensive, with the drug reimbursement list including all the 307 essential medicines and an increasing number of chemical and biologic medicines and traditional Chinese medicines [39,40].	Its drug coverage is comparatively fragmented and varies across different provinces since many provinces create their own separate and smaller list [41].	The drug coverage is comparatively fragmented and varies across different provinces since many provinces create their own separate and smaller list [41].	UEBMI’s general level of medical treatment is higher than URBMI and NCMS’s [29,38].
Reimbursement level	Higher reimbursement rate and reimbursement cap [29,32,43,44,45]; with 74.29–78.45% reimbursement rate of inpatient care [32], 72% comprehensive reimbursement rate [49].	Relatively lower reimbursement rate and reimbursement cap [29,32,43,44,45]; with 50.73–69.57% reimbursement rate of inpatient care [32], 50% comprehensive reimbursement rate [49].	The lowest reimbursement rate [29,32,43,44,45]; with 46.99–56.87% reimbursement rate of inpatient care [32], 40% comprehensive reimbursement rate [49].	NCMS’s reimbursement rate is higher in rural township hospitals than in municipal hospitals; with 84.04%, 61.25% and 47.71% reimbursement rate respectively at township, county and above medical institutions by the end of 2014 [46].

UEBMI = urban employee basic medical insurance. NCMS = new cooperative medical scheme. URBMI = urban resident basic medical insurance.

**Table 2:** Overview of dependent and independent variables.

Variables	Description	Mean	S.D.	Non-Missing Obs.
Outpatient service quality	Which type of medical facilities have you visited in the last month for outpatient treatment? 8 = General hospital; 7 = Specialized hospital; 6 = Chinese medicine hospital; 5 = Community healthcare center; 4 = Township hospital; 3 = Healthcare post; 2 = Village clinic/private clinic; 1 = Other. Higher score indicates higher quality.	4.8083	2.4687	4376
Conventional physical examination	How many items as follows do you take in the conventional checkup? Physical exam, routine blood test, routine urine test, lever function test, kidney function test, lipids profile test, blood glucose test, surgical, internal medicine, five sense organ test, electrocardiogram, B-type ultrasonic, chest fluoroscopy, male or female specialist, other. The number of chosen items is the score on this variable.	6.2154	4.3910	9040
Out-of-pocket expenditure	[The self-pay out-of-pocket expenditure for the medication in this visit]/[The total medication cost in this visit]	0.8162	0.6314	3854
[UEBMI: Yes→No and NCMS: Yes→Yes]	1 = if the insured person of UEBMI in 2013 turned out to be uninsured during 2013–2015, and meanwhile, this person participated in NCMS both in 2013 and during 2013–2015; 0 = otherwise.	0.2790	0.4486	6143
[UEBMI: Yes→No and NCMS: Yes→No]	1 = if the insured person of UEBMI in 2013 turned out to be uninsured during 2013–2015, and meanwhile, this person participated in NCMS in 2013 but did not during 2013–2015; 0 = otherwise.	0.5152	0.4998	6143
[UEBMI: Yes→No and NCMS: No→Yes]	1 = if the insured person of UEBMI in 2013 turned out to be uninsured during 2013–2015, and meanwhile, this person did not participate in NCMS in 2013 but did during 2013–2015; 0 = otherwise.	0.0493	0.2166	6143
[UEBMI: Yes→No and NCMS: No→No]	1 = if the insured person of UEBMI in 2013 turned out to be uninsured during 2013–2015, and meanwhile, this person did not participate in NCMS both in 2013 and during 2013–2015; 0 = otherwise	0.0656	0.2476	6143
[UEBMI: Yes→No and URBMI: Yes→Yes]	1 = if the insured person of UEBMI in 2013 turned out to be uninsured during 2013–2015, and meanwhile, this person participated in URBMI both in 2013 and during 2013–2015; 0 = otherwise.	0.0143	0.1189	17511
[UEBMI: Yes→No and URBMI: Yes→No]	1 = if the insured person of UEBMI in 2013 turned out to be uninsured during 2013–2015, and meanwhile, this person participated in URBMI in 2013 but did not during 2013–2015; 0 = otherwise	0.9476	0.2228	17511
[UEBMI: Yes→No and URBMI: No→Yes]	1 = if the insured person of UEBMI in 2013 turned out to be uninsured during 2013–2015, and meanwhile, this person did not participate in URBMI in 2013 but did during 2013–2015; 0 = otherwise	0.0012	0.0346	17511
[UEBMI: Yes→No and URBMI: No→No]	1 = if the insured person of UEBMI in 2013 turned out to be uninsured during 2013–2015, and meanwhile, this person did not participate in URBMI both in 2013 and during 2013–2015 0 = otherwise	0.0037	0.0608	17511

UEBMI = urban employee basic medical insurance. NCMS = new cooperative medical scheme. URBMI = urban resident basic medical insurance. Equal signs (=) indicate assigning scores to the respective variables.

**Table 3:** Overviews of demographic variables and the chronic diseases history.

**Variables**	**Panel A: Demographic Variables**		
	**Mean**	**S.D.**	**Non-Missing Obs.**
Age	59.4039	10.6551	20301
Gender [1 = Male, 2 = Female]	1.5241	0.4994	20899
**Variables**	**Panel B: The Chronic Diseases History**		
	**Frequency**		**Percentage %**
Hypertension [1 = Yes, 0 = No]	4190		20.04
Dyslipidemia [1 = Yes, 0 = No]	1770		8.47
Diabetes [1 = Yes, 0 = No]	1031		4.93
Cancer [1 = Yes, 0 = No]	198		0.95
Chronic lung diseases [1 = Yes, 0 = No]	1945		9.30
Liver disease [1 = Yes, 0 = No]	785		3.75
Heart disease [1 = Yes, 0 = No]	2155		10.31
Stroke [1 = Yes, 0 = No]	429		2.05
Kidney disease [1 = Yes, 0 = No]	1225		5.86
Stomach, or other digestive disease [1 = Yes, 0 = No]	4479		21.42
Emotional, nervous or psychiatric problem [1 = Yes, 0 = No]	265		1.27
Memory related disease [1 = Yes, 0 = No]	294		1.41
Arthritis or rheumatism [1 = Yes, 0 = No]	6507		31.12
Asthma [1 = Yes, 0 = No]	740		3.54

Equal signs (=) indicate assigning scores to the respective variables.

**Table 4:** The influence of urban unemployment-induced social health insurance transition (from UEBMI to NCMS) on healthcare utilization.

Variables	Dependent Variable: Healthcare Utilization								
	Outpatient Service Quality			Conventional Physical Examination			Out-of-Pocket Expenditure		
	Coef.	S.E.C	S.E.H	Coef.	S.E.C	S.E.H	Coef.	S.E.C	S.E.H
Unemployment-induced social health insurance transition									
[UEBMI: Yes→No and NCMS: Yes→Yes]	−1.1027 **	[0.2724]	[0.2665]	−3.3827 **	[0.3213]	[0.2867]	0.1596 **	[0.0469]	[0.0447]
[UEBMI: Yes→No and NCMS: Yes→No]	−0.2953	[0.2504]	[0.2498]	−1.6985 **	[0.3027]	[0.2668]	0.1015 *	[0.0446]	[0.0444]
[UEBMI: Yes→No and NCMS: No→Yes]	−0.8709 *	[0.3847]	[0.3884]	−2.1574 **	[0.4987]	[0.4677]	0.1448 *	[0.0573]	[0.0557]
[UEBMI: Yes→No and NCMS: No→No]	−0.8658 *	[0.3543]	[0.3599]	−2.9250 **	[0.4772]	[0.4269]	0.1011 ^†^	[0.0586]	[0.0581]
Demographic variables									
Gender	−0.4927 **	[0.1423]	[0.1500]	−0.7701 **	[0.1562]	[0.1574]	0.0197	[0.0218]	[0.0230]
Age	−0.0143 ^†^	[0.0074]	[0.0072]	−0.0182 *	[0.0086]	[0.0084]	−0.0063 **	[0.0011]	[0.0011]
The chronic disease history									
Hypertension	0.5042 **	[0.1761]	[0.1803]	0.0592	[0.2074]	[0.2021]	−0.0249	[0.0293]	[0.0292]
Dyslipidemia	0.4413 ^†^	[0.2289]	[0.2392]	0.5750 *	[0.2766]	[0.2711]	−0.0062	[0.0416]	[0.0382]
Diabetes	0.2175	[0.3084]	[0.2883]	0.6885 *	[0.3156]	[0.3224]	−0.0425	[0.0507]	[0.0483]
Cancer	−0.9128	[0.7573]	[0.7426]	−1.2926 ^†^	[0.6663]	[0.6998]	−0.0084	[0.0883]	[0.0907]
Chronic lung diseases	0.0373	[0.2396]	[0.2395]	0.6541 *	[0.3026]	[0.3052]	−0.0256	[0.0371]	[0.0384]
Liver disease	0.0143	[0.3170]	[0.3121]	0.0301	[0.3568]	[0.3861]	0.0435	[0.0379]	[0.0438]
Heart disease	0.5230 *	[0.2051]	[0.2121]	0.3443	[0.2717]	[0.2498]	−0.0087	[0.0367]	[0.0355]
Stroke	0.0805	[0.4144]	[0.4250]	−0.1345	[0.4838]	[0.4830]	−0.0243	[0.0653]	[0.0662]
Kidney disease	−0.1865	[0.3146]	[0.3025]	−0.2494	[0.3633]	[0.3813]	−0.0176	[0.0434]	[0.0404]
Stomach, or other digestive disease	−0.3369 *	[0.1710]	[0.1679]	−0.0624	[0.2067]	[0.2142]	0.0098	[0.0244]	[0.0250]
Emotional, nervous or psychiatric problem	0.6676	[0.5067]	[0.5112]	−1.7936 *	[0.8463]	[0.8513]	−0.0358	[0.0842]	[0.0871]
Memory related disease	0.5388	[0.4942]	[0.4798]	0.4220	[0.6039]	[0.5722]	−0.1012	[0.0814]	[0.0810]
Arthritis or rheumatism	−0.3152 ^†^	[0.1689]	[0.1684]	−0.8047 **	[0.1975]	[0.1941]	0.0533 *	[0.0255]	[0.0238]
Asthma	−0.3985	[0.3057]	[0.3217]	−0.2563	[0.4548]	[0.4798]	0.0238	[0.0472]	[0.0480]
Intercept term	7.2509 **	[0.5397]	[0.5418]	10.6938 **	[0.6196]	[0.6014]	1.0380 **	[0.0805]	[0.0827]
Observation size		1134	1134		2656	2656		1011	1011
Number of clusters that S.E. adjusted on		380	1075		431	2284		367	963
F statistics (*p*-value)		5.8300 (0.0000)	5.9600 (0.0000)		10.7300 (0.0000)	13.9400 (0.0000)		3.7300 (0.0000)	3.6300 (0.0000)

Robust standard errors (S.E.) have been clustered at community level and household level respectively (reported in the brackets). Coef. = estimated coefficient; S.E.C = robust S.E. on community level; S.E.H = robust S.E. on household level. UEBMI = urban employee basic medical insurance; NCMS = new cooperative medical scheme. ^†^ *p* < 0.10, * *p* < 0.05, ** *p* < 0.01.

**Table 5:** The influence of urban unemployment-induced social health insurance transition (from UEBMI to URBMI) on healthcare utilization.

	Dependent Variable: Healthcare Utilization								
	Outpatient Service Quality			Conventional Physical Examination			Out-of-Pocket Expenditure		
	Coef.	S.E.C	S.E.H	Coef.	S.E.C	S.E.H	Coef.	S.E.C	S.E.H
Unemployment-induced social health insurance transition									
[UEBMI: Yes→No and URBMI: Yes→Yes]	−1.2598 **	[0.4250]	[0.3868]	−2.2398 **	[0.4928]	[0.4719]	0.1952 **	[0.0524]	[0.0532]
[UEBMI: Yes→No and URBMI: Yes→No]	−1.2437 **	[0.2225]	[0.2232]	−2.9109 **	[0.2784]	[0.2354]	0.1588 **	[0.0438]	[0.0424]
[UEBMI: Yes→No and URBMI: No→Yes]	−1.3386	[0.9677]	[0.9706]	−0.9875	[1.8091]	[1.7795]	0.2712 *	[0.0450]	[0.0433]
[UEBMI: Yes→No and URBMI: No→No]	−1.2307	[1.4300]	[1.4249]	−1.3627 ^†^	[0.7117]	[0.7116]	0.1087	[0.1810]	[0.1776]
Demographic variables									
Gender	−0.3295 **	[0.0795]	[0.0816]	−0.8673 **	[0.0907]	[0.0904]	0.0245	[0.0251]	[0.0256]
Age	−0.0077 ^†^	[0.0044]	[0.0041]	−0.0088	[0.0058]	[0.0052]	−0.0030	[0.0018]	[0.0018]
The chronic disease history									
Hypertension	0.3891 **	[0.1040]	[0.1049]	−0.0893	[0.1127]	[0.1214]	−0.0320	[0.0220]	[0.0213]
Dyslipidemia	0.1994	[0.1477]	[0.1445]	0.6669 **	[0.1882]	[0.1712]	0.0076	[0.0330]	[0.0326]
Diabetes	0.1604	[0.1721]	[0.1682]	0.8714 **	[0.2104]	[0.2086]	−0.0390	[0.0290]	[0.0295]
Cancer	−0.1235	[0.3527]	[0.3639]	−0.1945	[0.4518]	[0.4740]	−0.0142	[0.0480]	[0.0476]
Chronic lung diseases	−0.2556 ^†^	[0.1300]	[0.1284]	0.1564	[0.1664]	[0.1782]	0.0125	[0.0385]	[0.0382]
Liver disease	−0.0646	[0.1926]	[0.1881]	0.0062	[0.2518]	[0.2554]	0.0046	[0.0270]	[0.0270]
Heart disease	0.2736 *	[0.1160]	[0.1281]	0.5098 **	[0.1797]	[0.1602]	−0.0199	[0.0239]	[0.0242]
Stroke	−0.0730	[0.2595]	[0.2592]	−0.2960	[0.3167]	[0.3056]	−0.0477	[0.0421]	[0.0426]
Kidney disease	0.0222	[0.1484]	[0.1456]	−0.2423	[0.2114]	[0.2045]	−0.0288	[0.0237]	[0.0230]
Stomach, or other digestive disease	−0.1505 ^†^	[0.0848]	[0.0913]	−0.1323	[0.1247]	[0.1198]	0.0021	[0.0215]	[0.0207]
Emotional, nervous or psychiatric problem	0.0691	[0.3196]	[0.3132]	−0.7203	[0.4382]	[0.3994]	−0.0428	[0.0510]	[0.0496]
Memory related disease	−0.0748	[0.3124]	[0.3032]	−0.0887	[0.4175]	[0.3875]	−0.0611	[0.0532]	[0.0547]
Arthritis or rheumatism	−0.2742 **	[0.0865]	[0.0884]	−0.5043 **	[0.1245]	[0.1122]	0.0310	[0.0284]	[0.0281]
Asthma	0.0058	[0.1878]	[0.1862]	−0.1565	[0.2856]	[0.2831]	−0.0381	[0.0353]	[0.0372]
Intercept term	6. 8319 **	[0.3654]	[0.3566]	10.2305 **	[0.4634]	[0.4088]	0.8313 **	[0.1306]	[0.1320]
Observation size		3552	3552		6778	6778		3142	3142
Number of clusters that S.E. adjusted on		429	3155		445	5313		426	2822
F statistics (*p*-value)		5.89 (0.0000)	5.90 (0.0000)		15.30 (0.0000)	19.49 (0.0000)		13.76 (0.0000)	15.16 (0.0000)

Robust standard errors (S.E.) have been clustered at community level and household level respectively (reported in the brackets). Coef. = estimated coefficient; S.E.C = robust S.E. on community level; S.E.H = robust S.E. on household level. UEBMI = urban employee basic medical insurance. URBMI = urban resident basic medical insurance. ^†^ *p* < 0.10, * *p* < 0.05, ** *p* < 0.01.

## Abbreviations

UEBMI	Urban employee basic medical insurance
URBMI	Urban resident basic medical insurance
NCMS	New cooperative medical scheme
CHARLS	China Health and Retirement Longitudinal Study
CNY	Chinese Yuan (currency)

## References

[1] Workers Job-Hop Every 22 Months on Average in China. http://www.sohu.com/a/225577892_114731.

[2] The Post-1995s Generation Leaves Office after 7 Months on Average in China. http://tech.ifeng.com/a/20180808/45108567_0.shtml.

[3] Wang H. (2009). A dilemma of Chinese healthcare reform: How to re-define government roles?. China Econ. Rev..

[4] Chen Q. (2018). The development of China’s basic medical insurance system and its main achievements. Chinese Research Perspectives on Population and Labor.

[5] Sun J., Deng S., Xiong X., Tang S. (2014). Equity in access to healthcare among the urban elderly in China: Does health insurance matter?. Int. J. Health Plan. Manag..

[6] Ye C., Duan S., Wu Y., Hu H., Liu X., You H., Wang L., Boog L., Dong H. (2013). A preliminary analysis of the effect of the new rural cooperative medical scheme on inpatient care at a county hospital. BMC Health Serv. Res..

[7] Yuan B., Li J., Wang Z., Wu L. (2019). Household registration system, migration, and inequity in healthcare access. Healthcare.

[8] Qiu P., Yang Y., Zhang J., Ma X. (2011). Rural-to-urban migration and its implication for new cooperative medical scheme coverage and utilization in China. BMC Public Health.

[9] Fan J.X., Wen M., Jin L., Wang G. (2013). Disparities in healthcare utilization in China: Do gender and migration status matter?. J. Fam. Econ. Issues.

[10] Li X., Zhang W. (2013). The impacts of health insurance on health care utilization among the older people in China. Soc. Sci. Med..

[11] Chen Y., Jin G.Z. (2012). Does health insurance coverage lead to better health and educational outcomes? Evidence from rural China. J. Health Econ..

[12] Hesketh T., Jun Y.X., Lu L., Mei W.H. (2008). Health status and access to health care of migrant workers in China. Public Health Rep..

[13] Lam K.K., Johnston J.M. (2012). Health insurance and healthcare utilisation for Shenzhen residents: A tale of registrants and migrants?. BMC Public Health.

[14] Peng Y., Chang W., Zhou H., Hu H., Liang W. (2010). Factors associated with health-seeking behavior among migrant workers in Beijing, China. BMC Health Serv. Res..

[15] Hong Y., Li X., Stanton B., Lin D., Fang X., Rong M., Wang J. (2006). Too costly to be ill: Health care access and health seeking behaviors among rural-to-urban migrants in China. World Health Popul..

[16] Zhao Y., Kang B., Liu Y., Li Y., Shi G., Shen T., Wang L. (2014). Health insurance coverage and its impact on medical cost: Observations from the floating population in China. PLoS ONE.

[17] You X., Kobayashi Y. (2009). The new cooperative medical scheme in China. Health Policy.

[18] Chen J. (2011). Internal migration and health: Re-examining the healthy migrant phenomenon in China. Soc. Sci. Med..

[19] Gong P., Liang S., Carlton E.J., Jiang Q., Wu J., Wang L., Remais J.V. (2012). Urbanisation and health in China. Lancet.

[20] Lin W., Liu G.G., Chen G. (2009). The urban resident basic medical insurance: A landmark reform towards universal coverage in China. Health Econ..

[21] Liu H., Zhao Z. (2014). Does health insurance matter? Evidence from China’s urban resident basic medical insurance. J. Comp. Econ..

[22] Huang F., Gan L. (2017). The impacts of China’s urban employee basic medical insurance on healthcare expenditures and health outcomes. Health Econ..

[23] Zhou Z., Zhu L., Zhou Z., Li Z., Gao J., Chen G. (2014). The effects of China’s urban basic medical insurance schemes on the equity of health service utilisation: Evidence from Shaanxi Province. Int. J. Equity Health.

[24] Yu B., Meng Q., Collins C., Tolhurst R., Tang S., Yan F., Bogg L., Liu X. (2010). How does the New Cooperative Medical Scheme influence health service utilization? A study in two provinces in rural China. BMC Health Serv. Res..

[25] Barber S.L., Yao L. (2010). Health Insurance Systems in China: A Briefing Note.

[26] Wang X., Zheng A., He X., Jiang H. (2014). Integration of rural and urban healthcare insurance schemes in China: An empirical research. BMC Health Serv. Res..

[27] Zhang L., Wang H. (2008). Dynamic process of adverse selection: Evidence from a subsidized community-based health insurance in rural China. Soc. Sci. Med..

[28] Pan J., Tian S., Zhou Q., Han W. (2016). Benefit distribution of social health insurance: Evidence from China’s urban resident basic medical insurance. Health Policy Plan..

[29] Meng Q., Fang H., Liu X., Yuan B., Xu J. (2015). Consolidating the social health insurance schemes in China: Towards an equitable and efficient health system. Lancet.

[30] Li C., Yu X., Butler J.R., Yiengprugsawan V., Yu M. (2011). Moving towards universal health insurance in China: Performance, issues and lessons from Thailand. Soc. Sci. Med..

[31] Liu K., Wu Q., Liu J. (2014). Examining the association between social health insurance participation and patients’ out-of-pocket payments in China: The role of institutional arrangement. Soc. Sci. Med..

[32] Pan Y., Chen S., Chen M., Zhang P., Long Q., Xiang L., Lucas H. (2016). Disparity in reimbursement for tuberculosis care among different health insurance schemes: Evidence from three counties in central China. Infect. Dis. Poverty.

[33] Chen G., Yan X. (2012). Demand for voluntary basic medical insurance in urban China: Panel evidence from the Urban Resident Basic Medical Insurance scheme. Health Policy Plan..

[34] Hou Z., Van de Poel E., Van Doorslaer E., Yu B., Meng Q. (2014). Effects of NCMS on access to care and financial protection in China. Health Econ..

[35] Zhang C.Y., Hashimoto H. (2015). How do patients and providers react to different incentives in the Chinese multiple health security systems?. Chin. Med. J..

[36] Zhou Z., Zhou Z., Gao J., Yang X., Yan J.E., Xue Q., Chen G. (2014). The effect of urban basic medical insurance on health service utilisation in Shaanxi Province, China: A comparison of two schemes. PLoS ONE.

[37] Sun Y., Gregersen H., Yuan W. (2017). Chinese health care system and Clin. Epidemiol. Clin. Epidemiol..

[38] Hu H., Chen J., Sato K.D., Zhou Y., Jiang H., Wu P., Wang H. (2016). Factors that associated with TB patient admission rate and TB inpatient service cost: A cross-sectional study in China. Infect. Dis. Poverty.

[39] Hu S. (2013). Essential medicine policy in China: Pros and cons. J. Med. Econ..

[40] Yip W.C.M., Hsiao W.C., Chen W., Hu S., Ma J., Maynard A. (2012). Early appraisal of China’s huge and complex health-care reforms. Lancet.

[41] Hu J., Mossialos E. (2016). Pharmaceutical pricing and reimbursement in China: When the whole is less than the sum of its parts. Health Policy.

[42] Huang Y., Jiang Y., Zhang L., Mao W., van Boven J.F., Postma M.J., Chen W. (2018). Availability, use, and affordability of medicines in urban China under universal health coverage: An empirical study in Hangzhou and Baoji. BMC Health Serv. Res..

[43] Liu X., Wong H., Liu K. (2015). Outcome-based health equity across different social health insurance schemes for the elderly in China. BMC Health Serv. Res..

[44] Yu H. (2015). Universal health insurance coverage for 1.3 billion people: What accounts for China’s success?. Health Policy.

[45] Jin Y., Hou Z., Zhang D. (2016). Determinants of health insurance coverage among people aged 45 and over in China: Who buys public, private and multiple insurance. PLoS ONE.

[46] Dai T., Hu H.P., Na X., Li Y.Z., Wan Y.L., Xie L.Q. (2016). Effects of new rural cooperative medical scheme on medical service utilization and medical expense control of inpatients: A 3-year empirical study of Hainan Province in China. Chin. Med. J..

[47] Chen B.K., Yang Y.T., Eggleston K. (2017). Patient copayments, provider incentives, and income effects: Theory and evidence from the essential medications list under China’s 2009 healthcare reform. World Med. Health Policy.

[48] Zhong H. (2011). Effect of patient reimbursement method on health-care utilization: Evidence from China. Health Econ..

[49] Meng Q., Tang S. (2013). Universal health care coverage in China: Challenges and opportunities. Procedia-Soc. Behav. Sci..

[50] Huang Y., Vemer P., Zhu J., Postma M.J., Chen W. (2016). Economic burden in Chinese patients with diabetes mellitus using electronic insurance claims data. PLoS ONE.

[51] Lin X., Cai M., Tao H., Liu E., Cheng Z., Xu C., Wang M., Xia X., Jiang T. (2017). Insurance status, inhospital mortality and length of stay in hospitalised patients in Shanxi, China: A cross-sectional study. BMJ Open.

[52] Liu H., Rizzo J.A., Fang H. (2015). Urban-rural disparities in child nutrition-related health outcomes in China: The role of hukou policy. BMC Public Health.

[53] Liu Z. (2005). Institution and inequality: The hukou system in China. J. Comp. Econ..

[54] Song Y. (2014). What should economists know about the current Chinese hukou system?. China Econ. Rev..

